# Association between tea consumption and depressive symptom among Chinese older adults

**DOI:** 10.1186/s12877-019-1259-z

**Published:** 2019-09-04

**Authors:** Ke Shen, Bin Zhang, Qiushi Feng

**Affiliations:** 1 School of Social Development and Public Policy, Fudan University, Shanghai, China; 2 Department of Sociology & Centre for Family and Population Research (CFPR), National University of Singapore, AS1 #04-30, 11 Arts Link, Singapore, 117570 Singapore

**Keywords:** Tea, Depressive symptom, Elderly, Chinese

## Abstract

**Background:**

Despite accumulating evidence on the protective effect of tea consumption against depression, studies specifically focusing on the elderly population are yet limited. This paper examined the association between the frequency and duration of tea drinking and depressive symptoms of older adults by gender and age groups, based on a nationally representative sample in China.

**Method:**

The study employed the panel data from 2005, 2008/2009, 2011/2012 and 2014 waves of Chinese Longitudinal Healthy Longevity Survey (CLHLS). We used the frequency and consistency of tea drinking behaviors to identify four types of tea consumption amongst Chinese seniors. Depressive symptoms were assessed by a five-item scale. Linear mixed effects models were applied.

**Results:**

We found that consistent and frequent tea-drinking was associated with significantly less depressive symptoms, and such impact was partially mediated by socioeconomic status, health behavior, physical health, cognitive function, and social engagement. However, the association was only significant for males and the oldest-old, rather than females and younger elders.

**Conclusions:**

Consistent and frequent tea-drinking may effectively reduce the risk of depressive symptoms for the Chinese elderly. The promotion of the traditional lifestyle of tea drinking could be a cost-effective way towards healthy aging for China.

**Electronic supplementary material:**

The online version of this article (10.1186/s12877-019-1259-z) contains supplementary material, which is available to authorized users.

## Background

Depression, as one of the most common mental disorders at old ages, often causes great suffering in later life. Unipolar depression or major depressive disorder, characterized by a persistent feeling of sadness or a lack of interest in outside stimuli, now affects about 7% of the older adults worldwide [1]. In China, the rising prevalence of depression amongst seniors has become a major health concern [2]. A recent nationwide survey in 2012, for example, reported that about one third of Chinese elderly aged 75 and over suffered severe depressive symptoms [3]. A growing body of research has been exploring risk factors for elderly depression, ranging from biomarkers, behavior characteristics, socioeconomic status, family structure, living arrangement, to community environment [4–9]. Amongst these factors, the consumption of tea, one of the most popular non-alcoholic beverages in the world, is nowadays drawing attention.

The positive impact of tea drinking on human health has been long acknowledged [10, 11]. According to a recent literature review [12], it has been well evidenced that tea consumption is inversely related to cancers of skin, prostate, lung and breast, as well as cardiovascular diseases (particularly atherosclerosis and coronary heart disease), diabetes, and arthritis. Another review paper [13] reported that the tea-drinking benefits on cardiovascular diseases should partially come from the improved nitric oxide status and endothelial function, whereas other possible pathways may work through body weight and fatness, oxidative damage, inflammation, platelet activation, blood pressure, and risk of Type-2 diabetes.

Researchers recently become interested in how tea drinking may promote mental health and some major biochemical mechanisms have been proposed [14]. Tea catechins, especially epigallocatechin gallate (EGCG), could exert antidepressant-like effects and prevent reduction in brain dopamine concentration [15, 16]. Theanine, one of the major amino acid contained in green tea leaves, could block the binding of L-glutamic acid and lead to lower post-stress cortisol and greater subjective relaxation [17]. Caffeine, which is an important component of tea, is well-known for increasing alertness and promoting mood [18]. Lastly, theaflavins as the main polyphenolic compounds of black tea may protect against oxidative stress [19]. Notwithstanding these important findings, how biochemical components interact to make up the benefit of tea upon mental health is still puzzling, and thus becomes a frontline in the current biochemical research for this field [20, 21].

The current epidemiological literature on the association between tea drinking and depression is still underdeveloped. Studies in this field mostly came from Asian societies, four from mainland China [19, 22–24], three from Japan [25–27], two from Taiwan [28, 29], one from South Korea [30], and one from Singapore [31]. Most of these studies focused on the elderly population. Based on these literatures, a recent meta-analysis provided supporting evidence for tea consumption against depression, though results in some studies as reviewed were yet to be insignificant [32]. Amongst studies specifically based on China, however, there has been no national-scaled research. In fact, to our knowledge, there are only two national-scaled investigations upon the health impact of tea drinking in China so far [33, 34], neither of which addressed the tea benefit over depression in older age.

In addition to the evident research gap in China, the current literature is also limited in a few other aspects. Regarding the protective role of tea against depression, the gender and age disparity in the effects of tea has not received enough attention in the field. Prior research showed that various risk factors of elderly depression, such as marital status, economic condition, disease burden and social support, may operate differently for men and women, for the young-old and the old-old [35–37]. Gender and age groups thus could be major moderators for the tea-depression association, which deserve more investigations [33]. Second, measurements of tea drinking habit in most studies only examined the frequency, amount, and types of tea consumption at the survey time or in the preceding month or year, without tracing the lifetime patterns, except one study [22]. This might be problematic in clarifying the causal relationship between tea consumption and depression, as the effect of tea could be long-termed. It is thus granted to incorporate better-refined measurements to address this issue.

Based on the panel data from 2005, 2008/2009, 2011/2012 and 2014 waves of Chinese Longitudinal Healthy Longevity Survey (CLHLS), the present study investigated the association between tea consumption and depressive symptoms among the Chinese older adults. To further contribute to the literature, we refined the measurement of tea drinking by incorporating the life experience of tea consumption, and examined the heterogeneity in associations between tea consumption and depressive symptoms by gender and by age groups.

## Methods

### Data source

The longitudinal data used in this study came from Chinese Longitudinal Healthy Longevity Survey (CLHLS). The CLHLS 1998 baseline survey interviewed 8959 oldest-old respondents aged 80 and over in randomly selected half of the counties in 22 out of 31 provinces in China [38]. Six waves of follow-up surveys were conducted in 2000, 2002, 2005, 2008/2009, 2011/2012 and 2014 respectively. The CLHLS has been expanded since 2002 to additionally include younger participants aged 65 to 79. The CLHLS systematically collected data from older adults through face-to-face interviews by trained staff. The data quality of the CLHLS is reasonably good in evaluations [39]. This study employed the longitudinal sample from 2005 to 2014 waves, which contained rich information on tea consumption and depressive symptoms of the respondents. After excluding cases with missing information on key variables, the final sample consisted of 13,026 elderly aged 65 and over interviewed in 2005 wave, with 5873 men (45.1%) and 7153 women (54.9%). The number of survived subjects remaining in follow-up surveys was 5458 in 2008–2009 wave, 3033 in 2011–2012 wave, and 1919 in 2014 wave.

### Measurement

To additionally capture the consistency of tea consumption in later years, we examined tea-drinking frequency of the interviewees at around age 60 as well as at the time of survey, with options of “almost every day”, “sometimes”, and “rarely or never”. Based on tea drinking frequency both in the past and at the present, we classified four types of tea drinkers. Those who rarely or never drank tea both at age 60 and at the time of survey were classified as “non-drinkers”, which was the reference category in the regression analyses. In contrast, those who drank tea almost every day both at age 60 and at the time of survey were defined as “consistent daily drinkers”. In between, those who drank tea at least sometimes at age 60 and at the time of survey, but not consistently daily, were “consistent drinkers”; and respondents who rarely or never drank tea either at age 60 or at the time of survey, but not both, were “inconsistent drinkers”.

Depressive symptoms in CLHLS were assessed by a five-item scale. These five items have been often adopted to indicate depressive symptoms in various studies using the CLHLS data [40–42]**.** Out of the five questions in this scale, two measured positive feelings and the other three measured negative affect. The former two questions included “do you look on the bright side of things?” and “are you as happy now as when you were young?”; and the other three questions were “do you often feel anxious or fearful?”, “do you often feel lonely and isolated?” and “do you feel the older you get the more useless you are?”. The respondents were requested to choose from five frequency responses of “Always”, “Often”, “Sometimes”, “Seldom” and “Never”. A score from 0 to 4 was assigned to each response, with a higher score indicating the higher frequency of feeling negative. In consequence, we had the summed score ranging from 0 to 20, with higher value indicating more depressive symptoms. The internal consistency reliability using Cronbach α coefficient based on the 2005 sample is 0.66, above the acceptable values of 0.6 [43]. Principle component analysis generated one factor with eigenvalues ≥ 1, explaining 44.5% of the total variance. The distribution of scores of depressive symptoms is presented in the Additional file 1: Figure S1.

We controlled for three groups of covariates that could have significant associations with elderly depression according to the prior studies [19, 44]. The first group included demographic and socioeconomic characteristics, including age, gender, residence (urban vs. rural), education (literate vs. illiterate), marital status (currently married vs. non-married), and pension status (with pension vs. without pension). The second group consisted of variable of lifestyle and health conditions, including current smoking (yes vs. no), current drinking (yes vs. no), activities of daily living (ADL) and cognitive function. ADL was based on whether the respondent could independently perform each of the six daily tasks, including feeding, bathing, dressing, toilet hygiene, continence and indoor transferring. The score of ADL ranges from 0 to 6, with higher scores indicating better functional independence. Cognitive function was measured using the Chinese version of Mini-Mental State Examination (MMSE), with a score ranging from 0 to 30 [45].

Lastly, we examined covariates of social engagement as the third group, which included activities such as playing card or mahjong (yes vs. no), participating community activities (yes vs. no), and tourism (yes vs. no). One key debate on the benefit of tea over mental health is whether the impact comes from the biochemical components of tea or the social context of tea drinking [21]. The confounding effect of social context should be particularly relevant in China, where tea drinking is a long cultural tradition with social functions to show respect, to build network, to celebrate, and to apologize. These covariates, which indicated stress-reducing leisure social activities for Chinese [46], could thus better clarify the biomedical and social aspects of the tea benefits.

### Analyses

Because responses on tea consumption, depressive symptoms and some covariates were time-varying for each respondent in the panel data, we employed linear mixed effect models, which could simultaneously and explicitly model the between- and within-individual variations. The model is expressed as:

1 $$ {Y}_{ij}={\beta}_0+{\beta}_1{T}_{ij}+{\beta}_2{Z}_{ij}+{b}_i+{\varepsilon}_{ij} $$

where *Y*_*ij*_ represents depressive symptoms for the *i*^*th*^ respondent at the *j*^*th*^ wave of survey. *T*_*ij*_ is the variable of tea consumption for the *i*^*th*^ respondent at the *j*^*th*^ wave of survey. *Z*_*ij*_ includes a series of covariates as mentioned in the previous section. *b*_*i*_ is the fixed effect of the *i*^*th*^ respondent, and *ε*_*ij*_ is the error term.

To clarify the effects of tea consumption on depressive symptoms, we followed a stepwise approach to adjust for different sets of mediating variables. Model 1 only included tea consumption habits. Model 2 added the demographic and socioeconomic covariates, such as age, gender, residence, education, pension status and marital status. Model 3 further controlled for the covariates of lifestyle and health conditions, including smoking, alcohol consumption, ADL and MMSE score. Model 4 further adjusted for variables of social engagement, including playing card or mahjong, participation in community activities and tourism. In order to examine the disparity in the association between tea drinking and depressive symptom by gender and age groups, we performed Model 4 separately for males and females, as well as for the young old and oldest-old.

## Results

Table 1 summarizes the characteristics of elderly respondents by types of tea drinkers in the 2005 wave of CLHLS. Of the 13,026 elderly participants, 38.1% were non-drinkers, 25.3% were inconsistent drinkers, 17.7% were consistent drinkers, and 18.9% were consistent daily drinkers. Compared with non-drinkers, drinkers, especially consistent daily drinkers, reported a significantly lower score of depressive symptoms (p < 0.001). The mean age, proportions of men and urban residents, and proportions of being educated, married and receiving pension, were also relatively higher among those who frequently and consistently consume tea. Meanwhile, tea drinkers tended to smoke and drink, but had better physical and cognitive functioning. And they were more socially involved.

**Table 1:** Characteristics of the elderly by tea consumption status in 2005 wave of CLHLS

	Non- drinkers	Inconsistent drinkers	Consistent drinkers	Consistent daily drinkers	*P* value
Depressive symptom, mean (SD)	6.92 (3.50)	6.72 (3.42)	6.59 (3.34)	5.86 (3.45)	< 0.001
Age, mean (SD)	85.95 (11.67)	85.15 (10.89)	84.07 (11.21)	82.13 (11.60)	< 0.001
Male, N (%)	1575 (31.75)	1493 (45.32)	1216 (52.66)	1589 (64.54)	< 0.001
Urban, N (%)	1991 (40.13)	1488 (45.17)	1084 (46.95)	1316 (53.45)	< 0.001
Educated, N (%)	1524 (30.72)	1402 (42.56)	1035 (44.82)	1449 (58.85)	< 0.001
Married, N (%)	1385 (27.92)	1100 (33.39)	801 (34.69)	1116 (45.33)	< 0.001
Having pension, N (%)	805 (16.23)	712 (21.62)	577 (24.99)	918 (37.29)	< 0.001
Smoking, N (%)	698 (14.07)	629 (19.10)	567 (24.56)	784 (31.84)	< 0.001
Drinking, N (%)	796 (16.05)	686 (20.83)	570 (24.69)	717 (29.12)	< 0.001
ADL score, mean (SD)	0.55 (1.29)	0.52 (1.28)	0.39 (1.14)	0.36 (1.09)	< 0.001
MMSE score, mean (SD)	23.63 (6.94)	24.04 (6.89)	24.93 (6.17)	25.96 (5.58)	< 0.001
Playing card/mahjong, N (%)	694 (13.99)	617 (18.73)	483 (20.92)	705 (28.64)	< 0.001
Community activity, N (%)	525 (10.58)	534 (16.21)	466 (20.18)	602 (24.45)	< 0.001
Tourism, N (%)	254 (5.12)	233 (7.07)	180 (7.80)	312 (12.67)	< 0.001
Sample size (%)	4961 (38.1)	3294 (25.3)	2309 (17.7)	2462 (18.9)	

*P* values are calculated with analysis of variance (ANOVA) for continuous variables and Chi-squared test for categorical variables

The regression results for associations between tea consumption and depressive symptoms are presented in Table 2. Model 1 shows that inconsistent and consistent tea drinkers had 0.22 and 0.35 point lower scores of depressive symptoms than non-drinkers respectively (both P < 0.01), whereas consistent daily drinkers had an even lower score of 0.95 point (P < 0.01). These raw outcomes support the inverse associations between tea consumption and depressive symptoms for Chinese old individuals. Once adjusting for demographic and socioeconomic traits (Model 2), the protective impacts of tea were attenuated. Only consistent daily drinkers maintained a significantly lower score of depressive symptoms (coefficient = 0.40, P < 0.01). This association was further mediated when variables of lifestyle and health condition (Model 3) and social engagement (Model 4) were added; however, consistent daily drinking remained a significant preventive factor against depressive symptoms (P < 0.01). These results reveal a strong protective role of tea consumption against depressive symptoms for Chinese elderly, though only when the drinking behavior was frequent and consistent.

**Table 2:** Associations between tea consumption and depressive symptoms for Chinese elderly (2005–2014 panel data)

	Model 1	Model 2	Model 3	Model 4
Inconsistent drinkers	− 0.22***	− 0.01	0.00	0.04
Consistent drinkers	−0.35***	−0.07	− 0.01	0.05
Consistent daily drinkers	− 0.95***	− 0.40***	− 0.31***	− 0.21***
Age		0.00	− 0.03***	− 0.03***
Male		−0.21***	0.05	0.03
Urban		−0.39***	−0.44***	− 0.38***
Literate		−0.46***	−0.35***	− 0.25***
Married		−0.77***	−0.76***	− 0.75***
Having pension		−0.82***	−0.74***	− 0.59***
Smoking			−0.14**	−0.11*
Drinking			−0.49***	−0.45***
ADL score			−0.29***	−0.27***
MMSE score			−0.09***	−0.08***
Playing card/mahjong				−0.48***
Community activity				−0.40***
Tourism				−1.00***
Cases	13,026	13,026	13,026	13,026
Observations	23,436	23,436	23,436	23,436

* *p* < 0.1, ** *p* < 0.05, *** *p* < 0.01

Results on covariates are also notable. Urban living, being educated and married, economic adequacy, better health and engagement in social activities were related to less depressive symptoms. It is worth noting that after controlling for health status, older age was associated with a significantly lower risk of depressive symptoms (Model 4). This result is indeed in line with the literature, probably due to decreased emotional responsiveness as well as increased emotional control and psychological immunization to stressful experiences with rising age [47, 48].

We further examined whether the tea benefits differ by gender and by age groups. As shown in Table 3, consistent daily tea consumption significantly lessened depressive symptoms for males (coefficient = − 0.29, P < 0.01), but not females; at the same time, the inverse association between consistent daily tea consumption and depressive symptoms was statistically significant for the young old (coefficient = − 0.42, P < 0.01), but not the oldest old aged 80 and above.

**Table 3:** Associations between tea consumption and depressive symptoms, by gender and by age group (2005–2014 panel data)

	By Gender		By Age group	
	Male	Female	80 and above	65–79
Inconsistent drinkers	0.03	0.02	0.08	−0.12
Consistent drinkers	−0.01	0.09	0.13	−0.09
Consistent daily drinkers	−0.29***	−0.09	− 0.01	−0.42***
Age	−0.02***	−0.04***	− 0.06***	0.02*
Male	–	–	0.06	−0.19**
Urban	−0.38***	−0.38***	− 0.42***	−0.31***
Literate	−0.43***	−0.03	− 0.24***	−0.16**
Married	−0.65***	−0.86***	− 0.52***	−0.89***
Having pension	−0.59***	−0.64***	− 0.62***	−0.69***
Smoking	−0.02	−0.28**	− 0.16**	0.00
Drinking	−0.40***	−0.52***	− 0.43***	−0.41***
ADL score	−0.37***	−0.22***	− 0.25***	−0.68***
MMSE score	−0.09***	−0.08***	− 0.08***	−0.10***
Playing card/mahjong	−0.30***	−0.69***	− 0.54***	−0.40***
Community activity	−0.26***	−0.57***	− 0.45***	−0.33***
Tourism	−1.09***	−0.88***	−0.95***	− 0.97***
Cases	5873	7153	8318	4708
Observations	10,895	12,541	13,992	9444

* *p* < 0.1, ** *p* < 0.05, *** *p* < 0.01

**Fig. 1:**
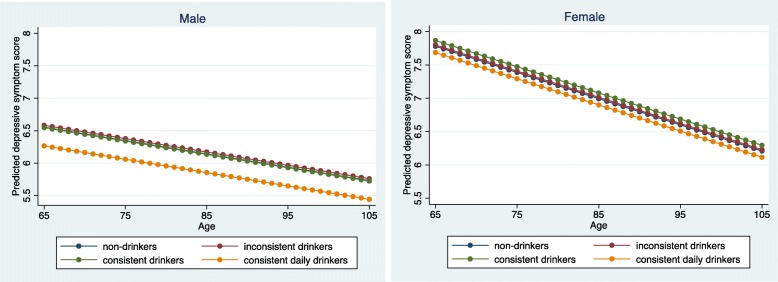
Average predicted depressive symptom scores for four groups of tea drinkers based on regression results, by gender and age. Note: All the covariates are set at their mean values

Figure 1 better illustrates the disparity by gender and age groups, which presents the predicted average depressive symptom scores of four types of tea drinkers, across the age range and by gender, with all covariates adjusted. As can be seen, at young ages, females suffered more depressive symptoms than males. With age advancement, the depressive symptom score decreased for both men and women, yet the slope was steeper for women. Regarding the impact of tea drinking, among male older adults, consistent daily drinkers reported much milder depressive symptoms than the other three groups; however, this pattern was not obvious for females.

## Discussion

With the worldwide population aging, the rising prevalence of mental disorders among older adults has gained increasing attentions in recent years. Based on the data from a nationwide longitudinal survey of Chinese elderly aged 65 and over, our study showed that consistent and frequent tea drinking effectively reduced depressive symptoms during the nine-year follow-up period. The results confirmed findings of previous epidemiological studies on the inverse association between tea drinking and depression amongst older adults [19, 22–31].

The study has major methodological strength. The linear mixed effects models as applied in this analysis could incorporate individual change over time, address the within-individual correlations, and thus provide more robust estimates of tea impact on mental health of Chinese seniors. More importantly, instead of examining tea-drinking habit at the time of survey or in the preceding month/year, we combined the information on frequency and consistency of tea consumption at age 60 and at the time of assessment, so that we developed a refined measurement classifying four types of tea drinkers. As tea-drinking behaviors possibly change in later years and the tea benefits might be long-termed, this two-time-point-based measure could be advantageous in capturing the role of tea consumption on mental health.

Using the refined measurement, our results revealed that only consistent daily drinkers, those who had drunk tea almost every day since age 60, could significantly benefit in mental health. The fact that inconsistent tea drinkers did not significantly mentally benefit suggests that the impact of tea may unfold in a long-termed manner. This finding contributes to the field, in which studies had been mostly focusing on the frequency of tea intake. In fact, current cohort prospective studies in the field tended to measure the tea-drinking habits at only one point of time and track the health impacts for a relatively short period of observation, gaining insignificant results [32]. The duration of tea drinking was recently noted to lower the incidence of Parkinson’s disease, hypertension, chronic gastritis and prostate cancer [49–52]. Along this line, this study highlighted the importance of consistent tea-drinking for mental health in later life.

Moreover, our results revealed that the inverse association between tea consumption and depressive symptoms was partially ameliorated by socioeconomic traits, health conditions, and participation in social activities. In this sense, through modeling the factors of social engagement, this study helped reveal the duality of benefits of tea drinking, both social and biochemical. This is not unexpected as tea drinking is a highly socialized behavior in China. In particular, the observed linkage between tea drinking and social engagement made good sense. In China as well as in many other Asian societies, tea drinking is not only a dietary habit, but a ceremonial symbol closely connected with various types of group events such as leisure activities and community events. Tea drinking is thus an indicator of active social participation, which helps lower the risk of depression [53, 54].

This study further examined the heterogeneity in associations between tea consumption and depressive symptoms by gender and age groups. We found that the benefit of tea drinking particularly applied to males and the young old, rather than females and the oldest old. This pattern is in line with a recent study conducted by Qiu and colleagues [33], where the benefits of tea against old-age mortality was particularly strong for young male elders aged 65 to 84 years, in contrast to oldest-old males and all females. Male gender and the younger age for the late life often indicate relatively better health than the females and oldest-old. It is likely that the benefit of tea is more evident for the early stage of heath deterioration and weakens for the late stage. According to a study on Finnish people, for example, tea intake was reported not effective in lowing the risk of severe depression [30]. Another possible reason is that men and younger elderly incline to drink more tea per day or drink stronger tea with a high concentration of beneficial constituents, and thus the tea benefit might be enlarged for these groups [19]. As discussed by Qiu and colleagues [33], there could be more biological and lifestyle mechanisms behind these interesting gender/age group disparities and further investigations are needed.

Despite these interesting findings above, cautions are surely needed in interpreting our results. Based on the large observational data from a national survey, the statistical association between tea drinking and depressive symptoms may not necessarily approve the clinical significance of tea components in improving mental health. This is particularly true given the fact that how tea drinking works involve both biochemical, behavioral and social mechanisms [21]. However, from the perspective of public health, in face of the accelerated population aging in China, our study suggests that it seems a reasonable initiative to promote tea drinking among seniors, which could effectively reduce depressive symptoms in later life. In particular, except for some high-end breeds, tea is usually affordable for most Chinese elderly in daily life. Thus, tea drinking is indeed cost-effective and easily adoptable, in regard to the maintenance and promotion of the elderly mental health. In addition, the benefit of tea is delivered not only through biomedical mechanisms but also in social realm, as drinking tea is often accompanied by social activities.

There are several major limitations to be noted in this study. First, we are not able to distinguish the type of tea and capture the exact quantity of tea intake due to lack of data. Without such information, accuracy of our estimation on the tea benefit over mental health would be compromised. Future studies are definitely needed to collect more information in this regard. Second, we acknowledge that the 5-item scale of depressive symptom is limited in comparison with the established scales of depression such as GDS or CES-D. Cautions are thus needed when interpreting the results. Last, although we discovered that tea consumption plays a much more important role in preventing depressive symptoms for males and the young old than for females and the oldest old, the underlying mechanisms are still speculative and more research are granted along this line.

## Conclusion

Given the increasing concern over disease burdens in China, our study offered a fresh perspective from dietary habits on how to advance mental health in an aging society. Consistent and frequent tea consumption, according to our study, was associated with significantly less depressive symptoms for Chinese old individuals, even adjusting for their socioeconomic status, lifestyle, health status and social engagement. In addition, the protective role of tea consumption was particularly strong for the males and younger elderly. Advocating for the traditional life habits such as tea drinking could be a promising way to promote healthy aging for China in the coming decades.

## Additional file

Additional file 1: **Figure S1** Distribution of Scores of Depressive Symptom (the 2005 wave of CLHLS) (DOCX 45 kb)

## Data Availability

The dataset is publicly available at https://sites.duke.edu/centerforaging/programs/chinese-longitudinal-healthy-longevity-survey-clhls/
