# Supplementary material for: Association between tea consumption and depressive symptom among Chinese older adults
Source: BMC Geriatr. 2019 Sep 4;19:246. doi: 10.1186/s12877-019-1259-z (PMC6724308; doi:10.1186/s12877-019-1259-z)
Supplement: Supplementary file 1 — Figure S1 Distribution of Scores of Depressive Symptom (the 2005 wave of CLHLS) (DOCX 45 kb) [file 12877_2019_1259_MOESM1_ESM.docx]

Additional file 1: Figure S1: Distribution of Scores of Depressive Symptom (the 2005 wave of CLHLS)
